# The comparative costs of implementing in-person and computerized interventions to enhance treatment receipt among drug-involved probationers

**DOI:** 10.1186/1940-0640-10-S1-A7

**Published:** 2015-02-20

**Authors:** Alexander J Cowell, Gary A Zarkin, Brendan Wedehase, Scott T Walters, Faye S Taxman

**Affiliations:** 1 Health, Social, and Economics Research, RTI International, Research Triangle Park, NC, 27703, USA; 2 Behavioral and Community Health, University of North Texas Health Science Center, Fort Worth, TX, 76107, USA; 3 Criminology, Law & Society, George Mason University, Fairfax, VA, 22030, USA

## Background

MAPIT is a three-arm randomized controlled trial assessing in-person Motivational Interviewing (MI) condition versus computerized (MAPIT) condition versus control. Our goal is to estimate the costs and cost-effectiveness for the study. We present preliminary findings on the startup costs and the implementation costs of the two intervention conditions, MI and MAPIT. To our knowledge, there is no published study of the cost-effectiveness of MI as a pretreatment intervention in a criminal justice setting. Similarly, there is no evidence to date on the cost-effectiveness of relevant web-based interventions in a criminal justice setting. An important contribution of this study is that it will address these significant gaps in the literature.

## Methods

The economic perspective—which determines whose costs are included—is that of the criminal justice system. The costs of the two interventions were assumed to be in addition to the control-arm costs. Implementation costs for both interventions were estimated by tracking the time interventionist staff screened, assessed, delivered, and supported each intervention. Interventionist staff kept timesheets on the time delivering and supporting each intervention. To estimate the study protocol cost per probationer in each intervention condition, a loaded hourly cost—including space and other relevant costs—was applied to that time. Costs incurred solely for research purposes were excluded.

## Results

Three preliminary findings on the average time that interventionists spent are: the time per probationer on appointment reminders for both the MI and MAPIT arms was 113 minutes; the time overseeing delivery of an MAPIT session was 21 minutes; and the time delivering an MI session was 53 minutes. The costs per protocol of MAPIT and MI were $76 and $137, respectively.

## Conclusions

Preliminary results suggest that using a computerized approach (MAPIT) to deliver the intervention is cheaper than using in-person counselors (MI). However, MAPIT costs were higher than anticipated, in part because of the relatively large amount of time spent on appointment reminders and overseeing delivery of that intervention. Before drawing any conclusions for clinical practice, it is important to combine costs and outcome data in cost-effectiveness analyses, which is a planned next step.

## Trial registration

NCT01891656

